# Cryptic biodiversity of tropical hesperiid caterpillar-attacking parasitoid wasps: three new species of *Creagrura* Townes (Hymenoptera, Ichneumonidae, Cremastinae) from Costa Rica and Perú

**DOI:** 10.3897/BDJ.10.e91486

**Published:** 2022-10-26

**Authors:** Ilari E. Sääksjärvi, Kari M. Kaunisto, Michael Sharkey, Shelby Stedenfeld, M. Alex Smith, Winnie Hallwachs, Daniel Janzen

**Affiliations:** 1 Biodiversity Unit, University of Turku, Turku, Finland Biodiversity Unit, University of Turku Turku Finland; 2 Department of Entomology, University of Kentucky, Lexington, Kentucky, United States of America Department of Entomology, University of Kentucky, Lexington Kentucky United States of America; 3 University of Kentucky, Department of Entomology, Kentucky, United States of America University of Kentucky, Department of Entomology Kentucky United States of America; 4 University of Guelph, Guelph, Canada University of Guelph Guelph Canada; 5 Department of Biology, University of Pennsylvania, Philadelphia, Philadelphia, United States of America Department of Biology, University of Pennsylvania, Philadelphia Philadelphia United States of America; 6 University of Pennsylvania, Philadelphia, United States of America University of Pennsylvania Philadelphia United States of America

**Keywords:** Área de Conservación Guanacaste, Allpahuayo-Mishana, Darwin wasps, Hesperiidae, koinobiont, Lepidoptera, Neotropical, nocturnal, rain forests

## Abstract

**Background:**

We describe three new species of the previously monotypic genus *Creagrura* Townes from Central and South America: *C.alejandromasisi* **sp. n.** and *C.rogerblancoi* **sp. n.** from Costa Rica and *C.allpahuaya* **sp. n.** from Peru, all of which emphasise the unknown parasitoid insect diversity yet to be revealed in the tropics.

**New information:**

Host relationships of the two Costa Rican species are described in detail. In addition, it is inferred that the *Creagrura* wasps find and oviposit in the caterpillar when it is exposed at night, rather than when it is concealed during daylight hours.

## Introduction

*Creagrura* Townes has been believed for 50 years to be a tropical monotypic genus of Cremastinae (Hymenoptera, Ichneumonidae) attacking caterpillars of Hesperiidae (Gauld 2007). The single known species, *C.nigripes*, was described by Townes (1971), based on adult specimens collected in Brazil, Ecuador and Suriname. Later, the taxonomy of the genus was briefly discussed by Gauld (2007).

*Creagrura* is structurally amongst the most distinguishable genera of cremastines. The females are easily separated from all other cremastines by the bizarre shape of their ovipositor, which is very short and strongly downcurved. The genus is also characterised by the broad ventral flanges of the mandibles and strongly raised lateral carinae of the scutellum. Despite the conspicuous appearance and interesting biology of the genus now revealed, it has remained little studied.

Gauld (2007) noted that, despite colour differences throughout the broad distribution of the monotypic genus (from Central America to Brazil as based on museum specimens and our samples), all the specimens appeared morphologically to be conspecific. He also noted considerable colour variation from Área de Conservación Guanacaste (ACG) (http://www.acguanacaste.ac.cr), north-western Costa Rica and commented to us (DHJ, WH) that he suspected that there might be several species within “*C.nigripes*” in ACG. The lack of strong morphological differences amongst “*C.nigripes*” may explain the lack of detailed studies of the genus. However, our findings, based on new material collected from Central and South America, suggest that the situation may be more complex.

During the last two decades, we (IES, KMK) have collected adult Darwin wasps (Ichneumonidae) in Peruvian Amazonia, for example, Sääksjärvi et al. (2004) and Gomez et al. (2018) and ACG has been the focus of 37 years of intense inventory of caterpillars and their parasitoids by DHJ and WH (Smith et al. 2006, Smith et al. 2008, Fernandez-Triana et al. 2014, Janzen and Hallwachs 2016, Sharkey et al. 2021). During the field works in Peru, we have discovered one distinctive new species of *Creagrura* from Amazonian lowland rain forests. On the other hand, based on hundreds of reared and barcoded ACG wasps and their host caterpillars, we (DHJ, WH) have discovered the existence of two additional cryptic species, each specialised to parasitise different genera of caterpillars feeding on two quite different groups of plants, morphologically and taxonomically (Poaceae + Cyperaceae vs. Marantaceae + Costaceae), side by side in lowland rain forest, but just one of them extending into adjacent dry forest. Both of the Costa Rican species of wasps were initially revealed by their different DNA barcodes.

Here, we describe these three new species. By describing these species, we add understanding to the enormous amount of cryptic and little-collected diversity of tropical koinobiont Darwin wasps being daily revealed by trapping and rearing of complex tropical ecosystems. Due to the restrictions of our research permits for Peruvian material, we have not been able to DNA barcode those *Creagrura* specimens.

## Materials and methods

Morphological terminology follows mainly that of Gauld (2007). Layer photos of all three new species were taken in the Biodiversity Unit, University of Turku, Finland (ZMUT), using an Olympus SZX16 stereomicroscope attached to a Canon 5DsR digital camera. Digital photos were combined using Helicon Focus Pro software. The holotype of *C.nigripes* was illustrated for comparison in the Townes Collection by Dr. David Wahl. The holotype of *C.nigripes* was compared with the new material by using those layer photos.

Sequence data, trace files and metadata for all the currently available two new species from ACG are available on BOLD (www.barcodinglife.org) using this DOI: https://dx.doi.org/10.5883.DS-ASCREAGR. Further samples after publication can be retrieved from BOLD by searching for individual voucher codes or the BIN code for each species (AAA2329, AAA5105). Currently, the two new ACG species are called *C.* nigripesDHJ01 and *C.* nigripesDHJ03 in these public databases. Additional collection information is deposited at http://janzen.sas.upenn.edu and all sequences have been deposited in the GenBank database, when they are transferred from BOLD to GenBank by the Centre for Biodiversity Genomics at the University of Guelph, Guelph, Canada.

The holotype and some paratypes of the Costa Rican species, which are formally Costa Rican government property on loan/custody to DHJ, are deposited on loan in the Utah State University (USU) insect collection, the new home for the former collection of the American Entomological Institute (AEI), previously located in Gainesville, Florida. Additional paratypes will be deposited in the Canadian National Insect collection in Ottawa (CNI) and in the Museo Nacional de Costa Rica (MNC), Santo Domingo de Heredia. A male and female paratype of each of the two new Costa Rican species are deposited in the ZMUT. The holotype and paratypes of the Peruvian species will be deposited in the Museo de Historia Natural, Universidad de San Marcos, Peru (UNSM). Two paratype females (Peruvian specimens) are in the ZMUT. The holotype and paratypes of the Peruvian species are currently on loan in ZMUT.

In the material examined, the SRNP code refers to the voucher code of the host caterpillar, while the DHJPAR code is the voucher code of the wasp itself, with full data available through http://janzen.sas.upenn.edu/caterpillars/database.lasso.

## Taxon treatments

### Creagrura

Townes, 1971

E411FA51-F410-5C7B-8989-28B7310CB76A

Creagrura Townes, 1971: 6. Type-species: *Creagruranigripes* Townes, by original designation.
Creagrura Creagrura nigripes Townes, 1971

#### Description

Modified from Gauld (2007).

Moderately large species, mainly yellowish-orange or orange-blackish, variously infuscate dorsally, front wings with a dark spot apically. Fore-wing length 8.0 to 9.8 mm. Clypeus separated from face by a suture. Mandibles with a broad ventral flange. Upper tooth of mandible longer and broader than lower tooth. Palpae formula 5:4. Frons slightly biconcave, polished. Antennae hirsute, flagellomeres infuscate, pedicel and scape variable in colouration. Occipital carina broadly interrupted mediodorsally, laterally strong, joining hypostomal carina at base of mandible. Pronotum unspecialised, with epomia slightly raised parallel to anterior margin, upper end detached and angled towards upper margin of pronotum. Mesoscutum with notauli present, broadly, but shallowly, depressed. Scutellum moderately convex, with strong lateral carinae reaching the posterior end. Mesopleuron smooth, punctuated on lower part. Epicnemial carina complete. Metapleuron punctated, separated from propodeum by a strong pleural carina. Propodeum with anterior and posterior transverse carinae present and complete. Lateral longitudinal carinae of propodeum present or rarely absent. Lateromedian longitudinal carinae present or rarely absent. Area superomedia more or less coffin-shaped or very rarely absent. Lateromedian longitudinal carina forming a V- or Y-shaped area basalis. Legs with tarsal claws small, pectinated to apices. Mid-tibia with two apical spurs. Hind femur smooth, without ventral tooth. Fore-wing with an enclosed oblique areolet. Pterostigma slender, blackish, narrower than first subdiscal cell. Distal abscissa of M complete to wing margin. Hind-wing with distal abscissae of M, Cu1 and 1A spectral distally or otherwise incomplete. Metasoma laterally strongly compressed. First tergite elongate, without glymma, ventral margins enclosing most of the sternite. Second tergite slender, varying in length, with a large thyridium. Laterotergite of the second tergite membranous, pendant. Ovipositor short and stout, orange-brownish in colouration and strongly decurved (hook-shaped), without subapical dorsal notch. Male claspers unspecialised, aedeagus slender, decurved, subapical bristles present.

#### Diagnosis

*Creagrura* is easy to distinguish from all other genera of the subfamily Cremastinae by the following set of characters: 1) ovipositor short and strongly down-curved, hook-shaped, 2) scutellum with strong lateral carinae, 3) mandible with broad ventral flange, 4) first tergite of metasoma ventrally almost completely enclosing the sternite and 5) second tergite of metasoma with a large thyridium.

#### Biology

In ACG, the two new species of *Creagrura* are middle to late instar koinobiont endoparasitoids of caterpillars that are diurnally concealed in longitudinally folded grass, sedge, ginger, palm or marantaceous leaves (Gauld 2007, DJ, WH, personal observation). However, this is a false impression created by the foraging habits of insect collectors. The caterpillars hide all day in a folded/rolled leaf of their food plant, but venture forth to eat that same and adjacent leaves at night. Since the wasps display all the yellow-orange colour and the behaviour of noctural adult Darwin wasps (Obs.: they do not have enlarged ocelli), we infer that the wasps find and oviposit in the caterpillar when it is exposed at night, rather than when it is concealed in a tight leaf tube during daylight hours. This hypothesis is further supported by the fact that specimens of *Creagrura* are rarely collected by Malaise trapping (see above) which is the standard tropical sampling method for diurnal Darwin wasps.

*Creagruraalejandromasisi* sp. n. (BIN AAA2329) is known only from ACG, where it is exclusively a specialist at parasiting the mid- to last instars of medium-sized (2-4 cm) Hesperiinae (Hesperiidae) caterpillars feeding on and day-time sequestering amongst the mature leaves of broad-leafed rain forest perenial monocots (Costaceae, Marantaceae, Cannaceae) in mostly insolated and full shade microhabitats 90-900 m elevation. It does not extend into adjacent ACG dry forest, as does *C.rogerblancoi* sp. n., which feeds on Poaceae, Arecaceae and Cyperaceae in both sun and shady microhabitats. It may be common elsewhere in Costa Rica, but not collected, simply because, in decades of Malaise trapping its ACG microhabitats, it has never been caught by a Malaise-trap. While there are many other genera and species of hesperiine and non-hesperiine caterpillars living and feeding in these microhabitats, *C.alejandromasisi* sp. n. is notable for parasitising only the following species of caterpillars (n = 634 of 155,932 ACG Hesperiidae wild caterpillars reared between 1978 and 2021), almost never a palm-eater, grass-eater or sedge-eater and 91% of the time reared from one of eight species of *Saliana* (Hesperiidae): (http://janzen.sas.upenn.edu/caterpillars/database.lasso): *Calpodesethlius* (5), *Cynea Burns02* (1), *Cyneairma* (2), *Cyneamegalops* (1), *Decineadecineaderisor* (1), *Parphoradecora* (1), *Rhinthonmolion* (1), *Rhinthonosca* (29), *Salianaantoninus* (70), *Saliana Burns03* (10), *Saliana Burns06* (1), *Salianaesperi* (379), *Salianafusta* (15), *Salianalongirostris* (1), *Salianaplacens* (3), *Salianaseverus* (85) and *Talides Burns04* (1). These caterpillars show a wide range of body types and colours, in contrast to those parasitised by *C.rogerblancoi*. An image of the solitary wasp cocoon with caterpillar cadaver is available at http://janzen.sas.upenn.edu/Wadults/searchplaycat4apr15.lasso?Voucher==05-SRNP-43145&-search and images of all of these species of caterpillars are available at htpp://janzen.sas.upenn.edu.

To date, *C.alejandromasisi* sp. n. has no suggestion of being attacked by any of the many tens of species of ACG common hyperparasitoids (e.g. *Mesochorus*, Ichneumonidae; *Perilampus*, Perilampidae, Chalcididae). Its host caterpillars are also attacked by a small array of other species of parasitoids, but those will be treated in other more cross-taxon ecological publications.

*Creagrurarogerblancoi* sp. n. (BIN AAA5105) has a caterpillar biology quite similar to that of *Creagruraalejandromasisi* sp. n. described above, except for its species of food plants and caterpillars, lesser sample size and a slight difference in sympatric microecosystems. While there are many other genera and species of hesperiine and non-hesperiine caterpillars living and feeding in its microhabitats, *C.rogerblancoi* sp. n. is notable for parasitising only the caterpillars of *Orsescynisca* (257), rarely three genera of grass-eating hesperiinae Hesperiidae and six species of *Perichares* (37) as grass-eating and understorey palm-eating caterpillars (306 of 155,932 ACG Hesperiidae wild caterpillars reared between 1978 and 2021). *Orsescynisca* is only feeding on leaves of four species of Cyperaceae and 34 species of Poaceae and the *Perichares* feed only on grasses and understorey palm leaves. *Creagruraalejandromasisi* never parasitises *Perichares* caterpillars feeding on palms or *Orsescynisca*, whatever plant species it is eating. Equally, *C.rogerblancoi* never attacks *Saliana* caterpillars, wherever they are feeding. As a result of their parasitisation of *Perichares* feeding on leaves of deeply shaded rainforest understorey palms, *C.rogerblancoi* wasps are more often reared from shady portions of the microhabitat than are *C.alejandromasisi*, but because the parasite-host interaction presumably takes place at night, this is probably just a serendipitous outcome of the species of host caterpillars and their food preferences. The caterpillars of the four species of *Perichares* studied intensively (Burns et al. 2008) are nearly identical in superficial appearance, but subtly different in their morphology to each other and similar to the caterpillars of *Orsescysnisca* (http://janzen.sas.upenn.edu/caterpillars/database.lasso). *C.rogerblancoi* is common throughout ACG lowland rainforest, but also extends into ACG lowland dry forest (parasitising hesperiine caterpillars eating grasses and sedges).

#### Taxon discussion

*Creagrura* Townes has been viewed as a monotypic Neotropical genus ranging from Central to South America for 50 years. Small morphological intraspecific variation previously led to the recognition of only one species, *C.nigripes* Townes 1971 (Townes 1971, Gauld 2007). Gauld (2007) stated that it is widespread throughout the Neotropics, but prior to more than a superficial understanding of ACG specimens and their DNA barcodes that emerged in 2004. This new understanding was not available to Gauld in 2007 because the indicative barcode data was still embedded in the gradually accumulating databases of the ACG inventory.

After studying a large amount of new genetic, morphological and biological data, it is now clear that there is more than one species in the genus and all previous descriptions of morphology and geographic ranges are pools of multiple species not individually recognised. All of the hundreds of the two new species, reared in the ACG biodiversity inventory, were misidentified as *Creagruranigripes*. There is no evidence that *C.nigripes* even occurs in Costa Rica or further north. Neither of the two new Costa Rican species have ever been captured in a Malaise trap, despite decades of Malaise trapping ACG forests where the two new species are very common. This result suggests that standard collections of tropical Hymenoptera are likely to not obtain *Creagrura*, despite its being a common wasp parasitising its common host species of Hesperiidae caterpillars. This note is further supported by our data from South America. Despite of collecting > 250 MTMs (Malaise trap months) in Peru (Gomez et al. 2018), we have found only a very few *Creagrura* specimens from these samples.

The mandibular flange and lateral carinae of the scutellum are key identifying characters of *Creagrura*, but they can also be found in some other Neotropical cremastines. Species of *Eiphosoma* Cresson exhibit this flange and one, apparently undescribed, Amazonian species has been found to possess a raised, lateral carina on the scutellum (Stedenfeld and Sääksjärvi, unpublished data).

### Creagrura alejandromasisi

Sääksjärvi, 2022 sp. n.

CA548CEF-DD8A-5B1D-A7F2-AFB7AE8C852A

F03E6918-80A2-4F71-B6BA-15ACC5CAA85A

#### Materials

- **Type status:** Holotype. **Occurrence:** individualID: 05-SRNP-43720; sex: Female; associatedSequences: DHJPAR0009786; occurrenceID: D2AE4CD4-686C-53BD-B042-39F54449B8A5; **Location:** continent: Americas; country: Costa Rica; locality: Area de Conservacion de Guanacaste; **Identification:** identifiedBy: D.H.Janzen, W.Hallwachs; **Event:** samplingProtocol: Rearing; **Record Level:** datasetID: DHJPAR0009786; institutionCode: AEI
- **Type status:** Paratype. **Occurrence:** individualID: 09-SRNP-40615; sex: Female; associatedSequences: DHJPAR0035191; occurrenceID: FFDD92D9-0F31-5DE3-B45F-60790C297B66; **Location:** continent: Americas; country: Costa Rica; locality: Area de Conservacion de Guanacaste; **Identification:** identifiedBy: D.H.Janzen, W.Hallwachs; **Event:** samplingProtocol: Rearing; **Record Level:** datasetID: DHJPAR0035191; institutionCode: CNI
- **Type status:** Paratype. **Occurrence:** individualID: 08-SRNP-564; sex: Female; associatedSequences: DHJPAR0023408; occurrenceID: F1733153-7631-5029-8D82-9887652D1283; **Location:** continent: Americas; country: Costa Rica; locality: Area de Conservacion de Guanacaste; **Identification:** identifiedBy: D.H.Janzen, W.Hallwachs; **Event:** samplingProtocol: Rearing; **Record Level:** datasetID: DHJPAR0023408; institutionCode: ZMUT
- **Type status:** Paratype. **Occurrence:** individualID: 08-SRNP-463; sex: Male; associatedSequences: DHJPAR0023423; occurrenceID: 4F44D56B-006C-5E89-928B-E063DD94CAF4; **Location:** continent: Americas; country: Costa Rica; locality: Area de Conservacion de Guanacaste; **Identification:** identifiedBy: D.H.Janzen, W.Hallwachs; **Event:** samplingProtocol: Rearing; **Record Level:** datasetID: DHJPAR0023423; institutionCode: AEI
- **Type status:** Paratype. **Occurrence:** individualID: 06-SRNP-34134; sex: Male; associatedSequences: DHJPAR0016378; occurrenceID: DC9AEF7C-E9B6-5CD6-A8B8-8C9830C964B4; **Location:** continent: Americas; country: Costa Rica; locality: Area de Conservacion de Guanacaste; **Identification:** identifiedBy: D.H.Janzen, W.Hallwachs; **Event:** samplingProtocol: Rearing; **Record Level:** datasetID: DHJPAR0016378; institutionCode: AEI
- **Type status:** Paratype. **Occurrence:** individualID: 08-SRNP-5087; sex: Male; associatedSequences: DHJPAR0028406; occurrenceID: 889FC6C5-7740-58B2-B622-E88B4B41CA10; **Location:** continent: Americas; country: Costa Rica; locality: Area de Conservacion de Guanacaste; **Identification:** identifiedBy: D.H.Janzen, W.Hallwachs; **Event:** samplingProtocol: Rearing; **Record Level:** datasetID: DHJPAR0028406; institutionCode: CNI
- **Type status:** Paratype. **Occurrence:** individualID: 08-SRNP-488; sex: Male; associatedSequences: DHJPAR0023422; occurrenceID: BE1B57F9-DAC9-5E9A-B00E-7FBB83E39296; **Location:** continent: Americas; country: Costa Rica; locality: Area de Conservacion de Guanacaste; **Identification:** identifiedBy: D.H.Janzen, W.Hallwachs; **Event:** samplingProtocol: Rearing; **Record Level:** datasetID: DHJPAR0023422; institutionCode: MNC
- **Type status:** Paratype. **Occurrence:** individualID: 07-SRNP-30407; sex: Male; associatedSequences: DHJPAR0017220; occurrenceID: 96AE0980-0B84-5250-A6D9-F790FAC7A5BD; **Location:** continent: Americas; country: Costa Rica; locality: Area de Conservacion de Guanacaste; **Identification:** identifiedBy: D.H.Janzen, W.Hallwachs; **Event:** samplingProtocol: Rearing; **Record Level:** datasetID: DHJPAR0017220; institutionCode: ZMUT

#### Description

Female: Mandibles with outer surface bearing long scattered whitish hairs; clypeus about 1.7-1.8 times as broad as high, strongly convex, shiny; lower face shiny, centrally with convex swelling (Fig. 1a). Mesoscutum polished, with median and lateral lobes punctate, posterior part of lateral lobe less punctate; notauli clearly impressed; meso- and metapleuron finely punctate, posterior transverse carina of the mesosternum strong and highly elevated. Propodeum in profile moderately long, extending to approximately 0.2-0.3 the length of posterior coxae, evenly declivous, with anterior and posterior transverse carinae strong, lateromedian longitudinal carina and pleural carina strong, lateral longitudinal carina more or less absent, weakly present posteriorly to posterior transverse carina and anteriorly to anterior transverse carina; area superomedia more or less coffin-shaped, curved laterally (Fig. 1d). Hind tarsal claw small, with a row of close pectinae. Metasoma with tergite 2 about 1.3 times as long as tergite 1, with a clearly discernible thyridium which is widely separated from the anterior margin, laterotergite membraneous, pendant (Fig. 1b). Ovipositor short, strongly de-curved, stout, without a subapical notch, ovipositor sheath with long, scattered hairs. Structure otherwise as figured (Fig. 1) and described in generic description.

A primarily yellowish species, with antenna, dorsal sripes (3) of pronotum, scutellum, propodeum, tarsal segments of mid-leg, tibia and tarsal segments of hind leg blackish or brownish; hind femur orange, with apical whitish spot; hind coxa orange; area dentipara brownish; metasoma predominantly orange, tergite 1 apically brownish, tergites 2-3 dorsally brownish or blackish (Fig. 1a). Ovipositor yellowish-orange, ovipositor sheath blackish. Wings hyaline, with veins, pterostigma and apical part (approximately 0.2) of front wing dark brownish.

Male: Similar to female in size, structure and colouration. Claspers simple, orange in colouration. aedeagus slender, slightly enlarged and rounded apically, with fine whitish britles apically.

#### Diagnosis

*Creagruraalejandromasisi* can be distinguished from other species of *Creagrura* by the following set of characters: tergite 1 about 1.3x as long as tergite 2; propodeum yellowish, with a brownish longitudinal stripe extending from area petiolaris to area basalis and brownish spot in area dentipara; hind coxa and femur orange and metasoma predominantly yellowish. It closely resembles the holotype of *C.nigripes* Townes in colouration. However, femurs of *C.nigripes* are shiny black and hind coxa orange ventrally and brownish dorsally. In addition, propodeum of *C.nigripes* has a W-shaped brown area dorsally. *C.alejandromasisi* sp. n. is sympatric (in Costa Rica) with *C.rogerblancoi* sp. n. However, these two species are readily distinguishable from each other by their colouration and their DNA barcodes.

#### Etymology

*Creagruraalejandromasisi* is named in honour of Costa Rica’s Alejandro Masís Cuevillas in recogntion of his 27 years of intense biological and administrative support to ACG parataxonomists, INBio, ACG as its Director and the Guanacaste Dry Forest Conservation Fund as its advisor.

#### Distribution

North-western Costa Rica.

#### Ecology

See above.

### Creagrura allpahuaya

Sääksjärvi, 2022 sp. n.

41203F0F-0F1C-5123-86AF-5175C9DFA0AB

E74B9CCD-9153-4ADE-943F-77459DF69836

#### Materials

- **Type status:** Holotype. **Occurrence:** sex: Female; occurrenceID: AE3C8A3C-96D5-53DA-9841-16BD70F6A5E9; **Location:** continent: South America; country: Peru; stateProvince: Loreto; locality: Allpahuayo; **Identification:** identifiedBy: Isrrael Gómez & Ilari E. Sääksjärvi; **Event:** samplingProtocol: Malaise trap; eventDate: 19-25.9-2011; habitat: Lowland rainforest; fieldNotes: Week18; **Record Level:** institutionCode: UNSM
- **Type status:** Paratype. **Occurrence:** sex: Female; occurrenceID: 74A3844F-5B54-5229-84FC-690142FC109B; **Location:** continent: South America; country: Peru; stateProvince: Loreto; locality: Allpahuayo; **Identification:** identifiedBy: Ilari E. Sääksjärvi et al.; **Event:** samplingProtocol: Malaise trap; eventDate: 14.9.-4.10.2000; habitat: Lowland rainforest, white sand; fieldNotes: APHIG3/1; **Record Level:** institutionCode: UNSM
- **Type status:** Paratype. **Occurrence:** sex: Female; occurrenceID: 714EC3C6-C904-5D7B-8D30-1863DD51723D; **Location:** continent: South America; country: Peru; stateProvince: Loreto; locality: Allpahuayo; **Identification:** identifiedBy: Ilari E. Sääksjärvi (IES) & Reijo Jussila (RJ); **Event:** samplingProtocol: Malaise trap; eventDate: 18.9.-4.10.1998; habitat: Lowland rainforest, white sand (varillal); fieldNotes: APHI D1/3 012; **Record Level:** institutionCode: UNSM
- **Type status:** Paratype. **Occurrence:** sex: Female; occurrenceID: 9A666798-1999-5A7D-BF68-C92B3930ACF3; **Location:** continent: South America; country: Peru; stateProvince: Loreto; locality: Allpahuayo; **Identification:** identifiedBy: Ilari E. Sääksjärvi (IES) & Reijo Jussila (RJ); **Event:** samplingProtocol: Malaise trap; eventDate: 2-24.3.2000; habitat: Lowland rainforest, white sand; fieldNotes: APHI G1/3; **Record Level:** institutionCode: UNSM
- **Type status:** Paratype. **Occurrence:** sex: Female; occurrenceID: 2D8F44FE-9F21-5F8D-A3B5-C82708144023; **Location:** continent: South America; country: Peru; stateProvince: Loreto; locality: Allpahuayo; **Identification:** identifiedBy: Ilari E. Sääksjärvi et al.; **Event:** samplingProtocol: Malaise trap; eventDate: 1-15,12.2000; habitat: Lowland rainforest, clay; fieldNotes: APHI J1/17; **Record Level:** institutionCode: ZMUT
- **Type status:** Paratype. **Occurrence:** sex: Male; occurrenceID: E6D28160-38B1-53C4-9BFD-ABB906D955BD; **Location:** continent: South America; country: Peru; stateProvince: Loreto; locality: Mishana; **Identification:** identifiedBy: Ilari E. Sääksjärvi (IES) & Reijo Jussila (RJ); **Event:** samplingProtocol: Malaise trap; eventDate: 1-16.11.1998; habitat: Lowland rainforest, clay; fieldNotes: APHI A1/6 022; **Record Level:** institutionCode: UNSM

#### Description

Female: Mandibles with outer surface bearing long scattered whitish hairs; clypeus about 1.7-1.8 times as broad as high, strongly convex, shiny; lower face shiny, centrally with convex swelling (Fig. 2c). Mesoscutum polished, with median and lateral lobes punctate, posterior part of lateral lobe less punctate; notauli clearly impressed; meso- and metapleuron finely punctate, posterior transverse carina of the mesosternum strong and highly elevated. Propodeum in profile very long, extending to approximately 0.4-0.5x the length of posterior coxae, evenly declivous, with anterior and posterior transverse carinae strong, lateromedian longitudinal carina and pleural carina strong, lateral longitudinal carina more or less absent, weakly present posteriorly to posterior transverse carina and anteriorly to anterior transverse carina; area superomedia more or less coffin-shaped, straight laterally (Fig. 2d). Hind tarsal claw small, with a row of close pectinae. Metasoma with tergite 2 about 1.6 times as long as tergite 1, with a clearly discernible thyridium which is widely separated from the anterior margin, laterotergite membraneous, pendant (Fig. 2b). Ovipositor short, strongly de-curved, stout, without a subapical notch, ovipositor sheath with long, scattered hairs. Structure otherwise as figured (Fig. 2) and described in generic description.

A primarily yellowish-orange species, with antenna, hind femur, hind tibia, hind tarsal segments and tergites 2-6 dark brownish to shiny blackish; central lobe of pronotum brownish; propodeum orange, without brownish areas. Ovipositor dark brown or orange, ovipositor sheaths blackish. Wings hyaline, with veins, pterostigma and apical part (approximately 0.2) of front wing blackish. Structure otherwise as figured (Fig. 2) and described in generic description.

Male: Similar to female in size, structure and colouration. Claspers simple, brownish in colouration. Aedeagus slender, slightly enlarged and rounded apically, with fine whitish britles apically. Obs.: only one male specimen has been found. This specimen has strongly elevated propodeal carination.

#### Diagnosis

*Creagruraallpahuaya* sp. n. is the most distinctive species of *Creagrura*. It can be easily distinguished from other species of *Creagrura* by the following set of characters: tergite 2 long, approximately 1.6 times as long as tergite 1, mesosoma predominatly yellowish-orange, hind coxa orange (ventrally and laterally), hind femur (except for apical white spot), tibia and tarsal segments black and tergite 2-6 predominantly blackish. It resembles *C.nigripes* in colouration of hind legs (both species have black or blackish hind femura). However, metasoma of *C.nigripes* is predominantly orange and it has large brownish areas in pronotum, scutellum, propodeum and hind coxa. These structures are yellowish-orange in *C.allpahuaya*.

#### Etymology

The specific name “*allpahuaya*” refers to the type locality: National Reserve of Allpahuayo-Mishana (Perú).

#### Distribution

Peru.

#### Biology

Nothing is known about the host relationships of this species.

#### Taxon discussion

This species is only known from the Western Amazonian lowland rain forests (National Reserve of Allpahuayo-Mishana, Department of Loreto, Iquitos, Perú).

### Creagrura rogerblancoi

Sääksjärvi, 2022 sp. n.

F086DD7B-87FD-5C10-BEBE-A5E8C95272EA

45235D41-927F-4229-AE3D-32301B2EC9CF

#### Materials

- **Type status:** Holotype. **Occurrence:** individualID: 06-SRNP-43689; sex: Female; associatedSequences: DHJPAR0016381; occurrenceID: 0658998C-FA34-5B6E-BDC2-A5E0BCE03F70; **Location:** country: Costa Rica; locality: Area de Conservacion de Guanacaste; **Identification:** identifiedBy: D.H.Janzen & W. Hallwachs; **Event:** samplingProtocol: Rearing; **Record Level:** institutionCode: AEI
- **Type status:** Paratype. **Occurrence:** individualID: 08-SRNP-57407; sex: Female; associatedSequences: DHJPAR28409; occurrenceID: BE2D3220-3903-56A9-8F71-9A8098ED7C86; **Location:** country: Costa Rica; locality: Area de Conservacion de Guanacaste; **Identification:** identifiedBy: D.H.Janzen & W. Hallwachs; **Event:** samplingProtocol: Rearing; **Record Level:** institutionCode: AEI
- **Type status:** Paratype. **Occurrence:** individualID: 07-SRNP-42145; sex: Female; associatedSequences: DHJPAR0021134; occurrenceID: 60A782D4-A10E-53C8-8FAC-A8C3062E046E; **Location:** country: Costa Rica; locality: Area de Conservacion de Guanacaste; **Identification:** identifiedBy: D.H.Janzen & W. Hallwachs; **Event:** samplingProtocol: Rearing; **Record Level:** institutionCode: CNI
- **Type status:** Paratype. **Occurrence:** individualID: 07-SRNP-42145; sex: Female; associatedSequences: DHJPAR0021134; occurrenceID: 49EAE486-0DC5-53FB-8D9C-3BDE8133A564; **Location:** country: Costa Rica; locality: Area de Conservacion de Guanacaste; **Identification:** identifiedBy: D.H.Janzen & W. Hallwachs; **Event:** samplingProtocol: Rearing; **Record Level:** institutionCode: CNI
- **Type status:** Paratype. **Occurrence:** individualID: 08-SRNP-57409; sex: Female; associatedSequences: DHJPAR0028410; occurrenceID: 80375DD1-D09D-5A10-AA90-95FC4663F1C9; **Location:** country: Costa Rica; locality: Area de Conservacion de Guanacaste; **Identification:** identifiedBy: D.H.Janzen & W. Hallwachs; **Event:** samplingProtocol: Rearing; **Record Level:** institutionCode: MNC
- **Type status:** Paratype. **Occurrence:** individualID: 07-SRNP-40196; sex: Female; associatedSequences: DHJPAR0016933; occurrenceID: 9C3FECC4-CAE3-5CB1-8BE5-A96E60F3C4C8; **Location:** country: Costa Rica; locality: Area de Conservacion de Guanacaste; **Identification:** identifiedBy: D.H.Janzen & W. Hallwachs; **Event:** samplingProtocol: Rearing; **Record Level:** institutionCode: ZMUT
- **Type status:** Paratype. **Occurrence:** individualID: 07-SRNP-42148; sex: Male; associatedSequences: DHJPAR0021104; occurrenceID: 77A35467-6A2C-5FE1-A749-FE086087D8FC; **Location:** country: Costa Rica; locality: Area de Conservacion de Guanacaste; **Identification:** identifiedBy: D.H.Janzen & W. Hallwachs; **Event:** samplingProtocol: Rearing; **Record Level:** institutionCode: AEI
- **Type status:** Paratype. **Occurrence:** individualID: 07-SRNP-56455; sex: Male; associatedSequences: DHJPAR0019860; occurrenceID: 191EB5B2-488A-5B8F-9E66-29833F5779A5; **Location:** country: Costa Rica; locality: Area de Conservacion de Guanacaste; **Identification:** identifiedBy: D.H.Janzen & W. Hallwachs; **Event:** samplingProtocol: Rearing; **Record Level:** institutionCode: AEI

#### Description

Female: Mandibles with outer surface bearing long scattered whitish hairs; clypeus about 1.7-1.8 times as broad as high, strongly convex, shiny; lower face shiny, centrally with convex swelling (Fig. 3c). Mesoscutum polished, with median and lateral lobes punctate, posterior part of lateral lobe less punctate; notauli clearly impressed; meso- and metapleuron finely punctate, posterior transverse carina of the mesosternum strong and highly elevated. Propodeum in profile moderately long, extending to approximately 0.2-0.3x the length of posterior coxae, evenly declivous, with anterior and posterior transverse carinae strong, lateromedian longitudinal carina and pleural carina strong, lateral longitudinal carina more or less absent, weakly present posteriorly to posterior transverse carina and anteriorly to anterior transverse carina; area superomedia more or less coffin-shaped, straight laterally (Fig. 3d). Hind tarsal claw small, with a row of close pectinae. Metasoma with tergite 2 about 1.3 times as long as tergite 1, with a clearly discernible thyridium which is widely separated from the anterior margin, laterotergite membraneous, pendant (Fig. 3b). Ovipositor short, strongly de-curved, stout, without a subapical notch, ovipositor sheath with long, scattered hairs. Structure otherwise as figured (Fig. 3) and described in generic description.

A primarily yellowish-orange species, with antenna, hind tibia, hind tarsal segments, tergite 1 dorsally and apical part of tergite 2 dorsally black or blackish; central lobe of pronotum fuscous; propodeum orange, without brownish areas; hind femur orange, with apical whitish spot; hind coxa orange (Fig. 3b). Ovipositor yellowish-orange, ovipositor sheaths blackish. Wings hyaline, with veins, pterostigma and apical part (approximately 0.2) of front wing blackish. Structure otherwise as figured (Fig. 3) and described in generic description.

Male: Similar to female in size, structure and colouration. Claspers simple, orange in colouration. aedeagus slender, slightly enlarged and rounded apically, with fine whitish britles apically.

#### Diagnosis

*Creagrurarogerblancoi* sp. n. can be distinguished from other species of *Creagrura* by the following set of characters: tergite 1 about 1.3x as long as tergite 2, propodeum entirely orange (without any brownish or blackish spots or stripes), hind coxa orange (both ventrally and dorsally) and hind femur orange. This species is sympatric (in Costa Rica) with *C.alejandromasisi* sp. n. However, these two species are readily distinguishable by their colouration. *C.rogerblancoi* is also readly distinguished from *C.nigripes* which has, for example, blackish hind femurs and brownish “W-shape” on propodeum.

#### Etymology

This species is named in honour of Costa Rica’s Roger Blanco Segura to honour his four decades of intense and dedicated management and care of the biodiversity of Parque Nacional Santa Rosa and then its expansion to become Área de Conservación Guanacaste, Costa Rica.

#### Distribution

North-western Costa Rica.

#### Biology

See above.

## Discussion

### General patterns in species richness of Neotropical parasitoid wasps

Darwin wasps (Hymenoptera, Ichneumondiae) have been hypothesised to be more species-poor in the tropics than in some higher latitudes (Gauld 1991, Gauld et al. 1992, Timms et al. 2016), but this is obviously a misconception, based on limited classical ways of insect collecting and on relying on morphological differentiation of museum specimens with no appreciation of their natural history.

Recent thorough trapping and host rearing in ACG habitats and Peruvian Amazonia (Sääksjärvi et al. 2004, Smith et al. 2008, Veijalainen et al. 2012, Gomez et al. 2018, Janzen et al. 2020, Sharkey et al. 2021,) are revealing that there are enormous numbers of species of Neotropical parasitoid wasps that are unappreciated and unknown to science and society.

The three described here are emphasised now because their roles in emerging ACG ecological patterns need identifying names and because they are readily distinctive from what was believed to be the single species in a monotypic genus.

## Supplementary Material

XML Treatment for Creagrura

XML Treatment for Creagrura alejandromasisi

XML Treatment for Creagrura allpahuaya

XML Treatment for Creagrura rogerblancoi

## Figures and Tables

**Figure 1a.**
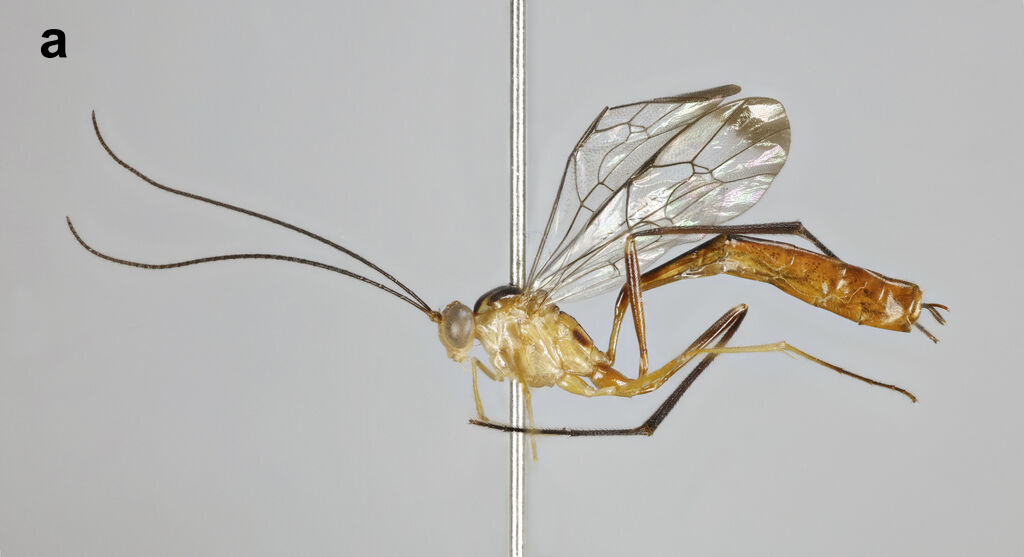
Holotype, female: habitus (lateral)

**Figure 1b.**
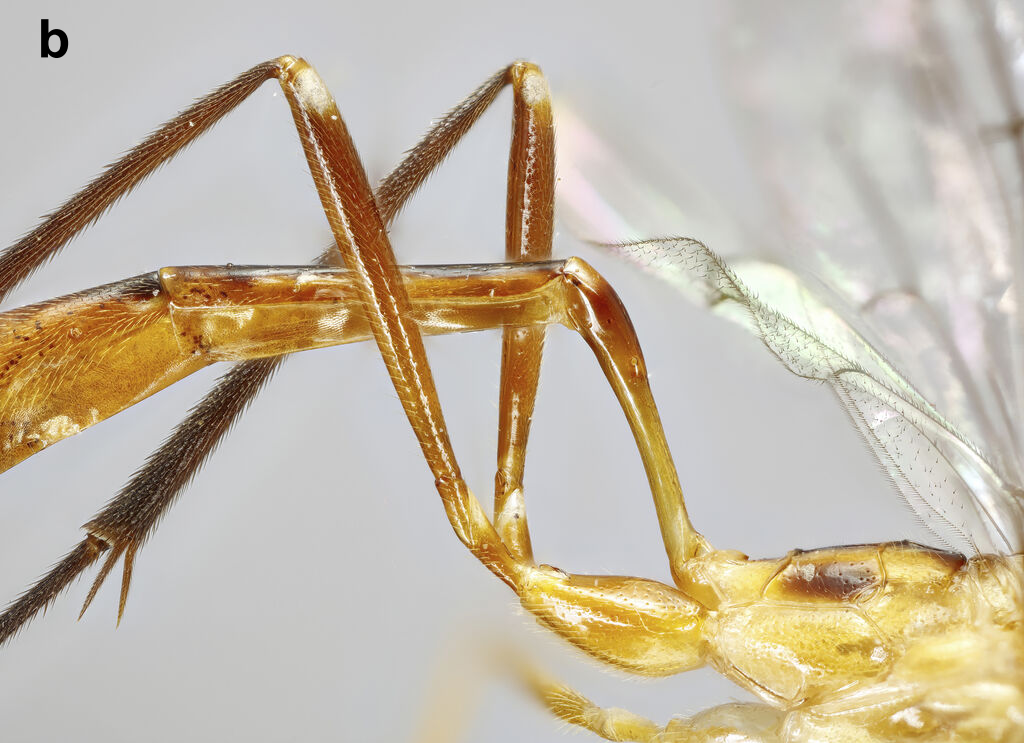
Paratype, female: first and second tergites (lateral)

**Figure 1c.**
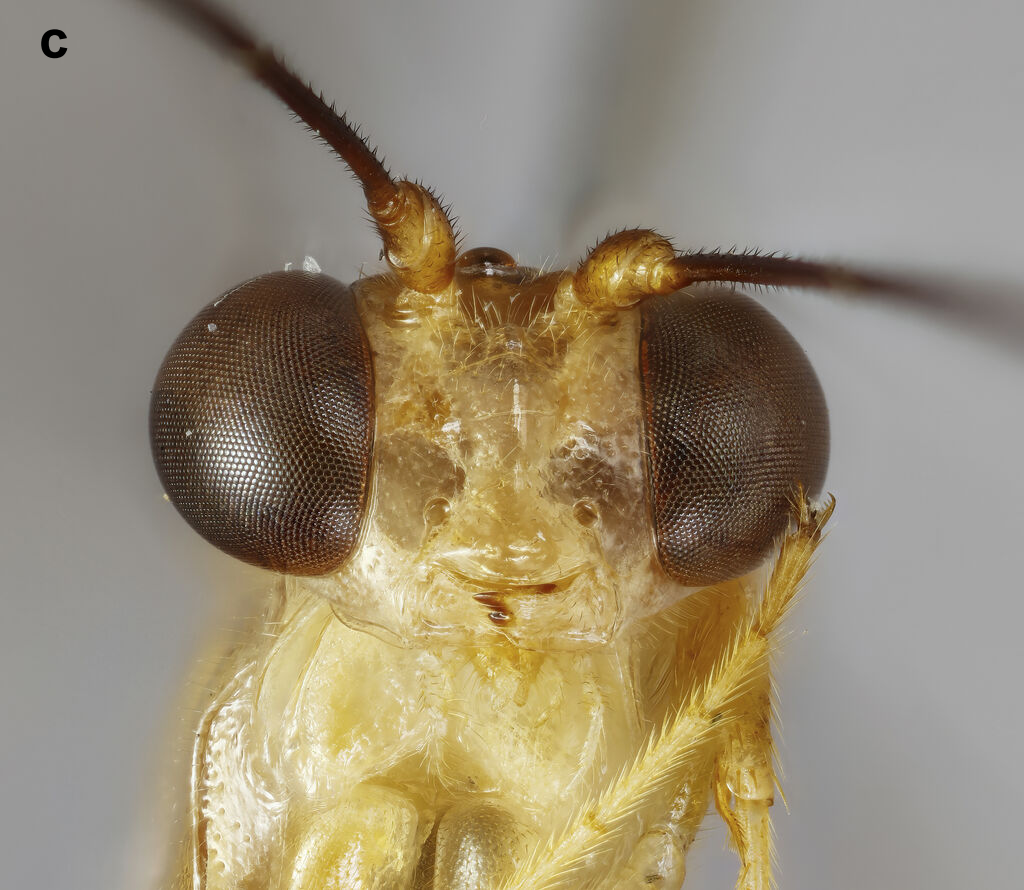
Paratype, female: face (anterior)

**Figure 1d.**
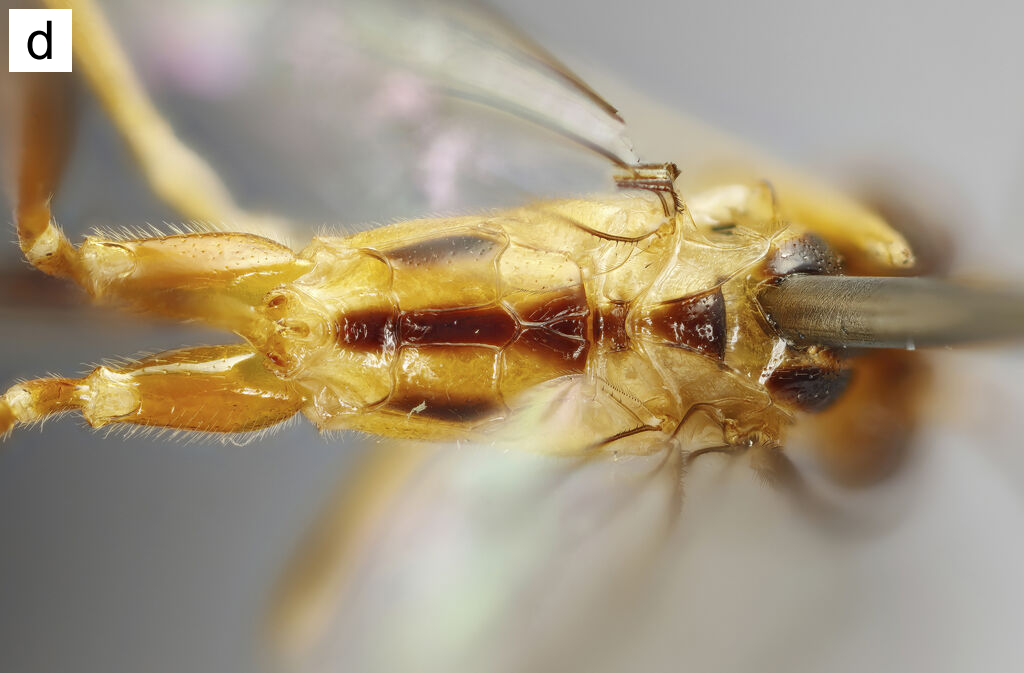
Paratype, female: propodeum (dorsal)

**Figure 2a.**
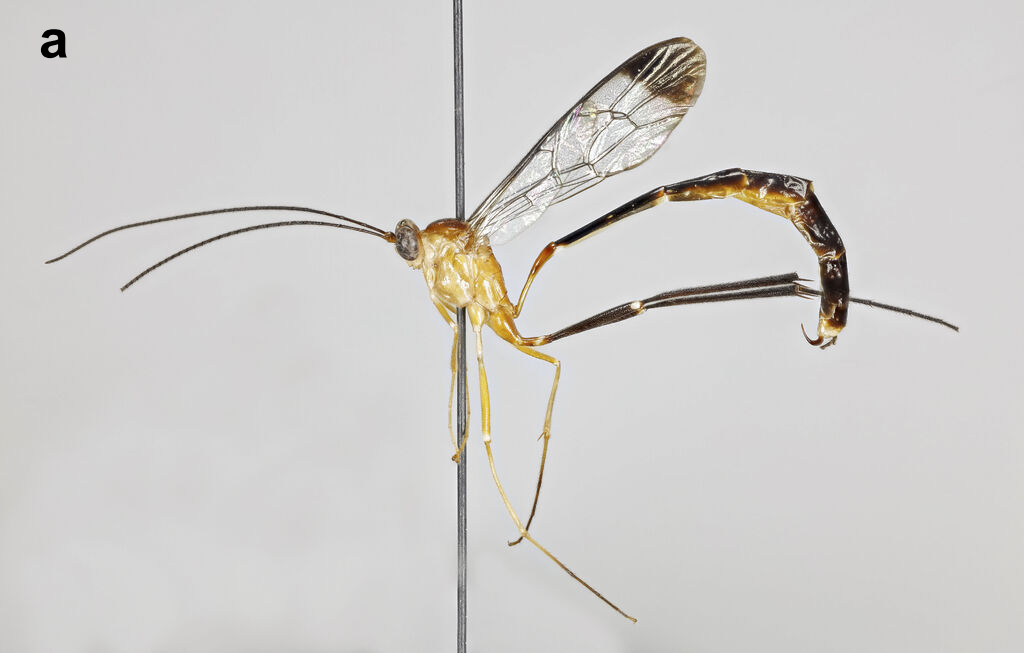
Habitus (lateral)

**Figure 2b.**
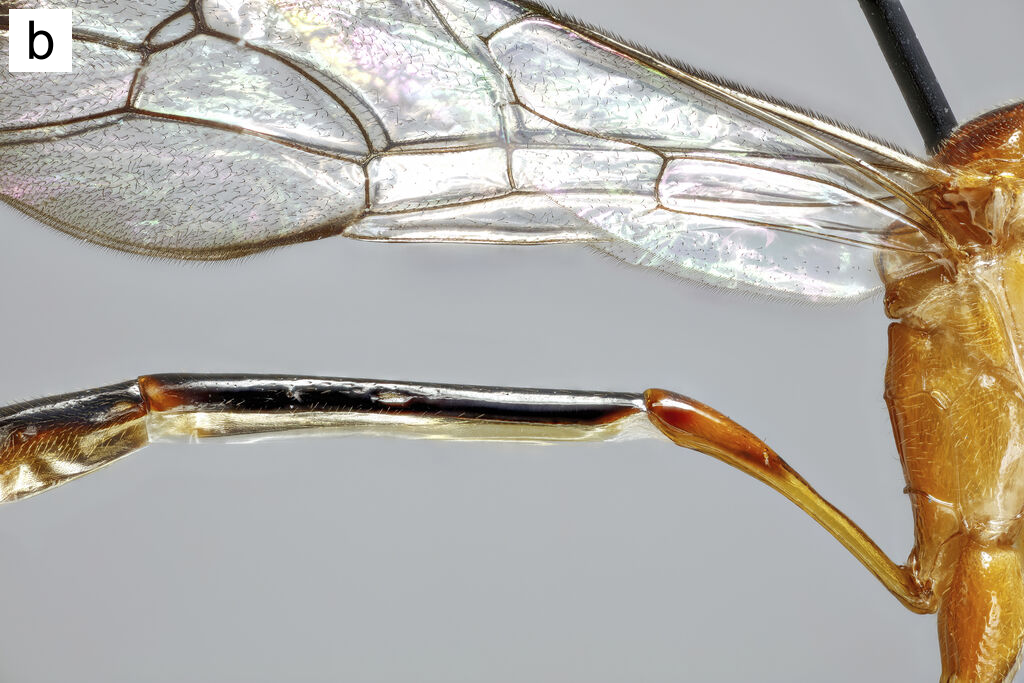
First and second tergites (lateral)

**Figure 2c.**
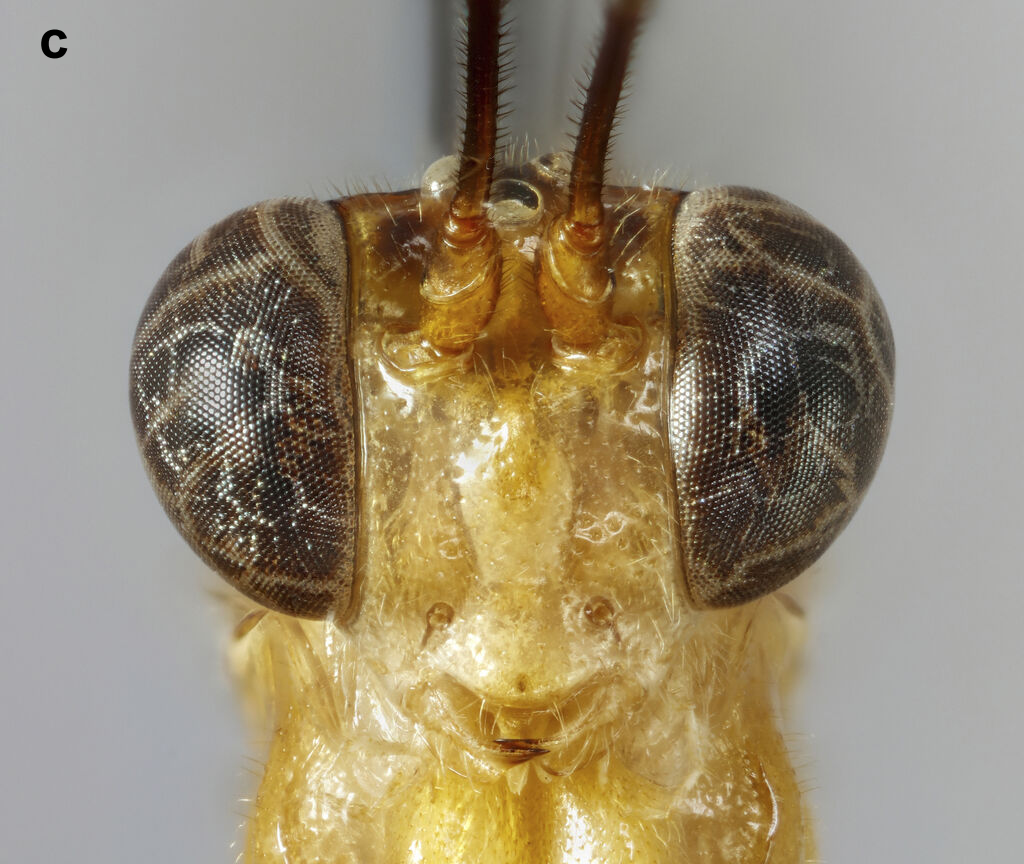
Face (anterior)

**Figure 2d.**
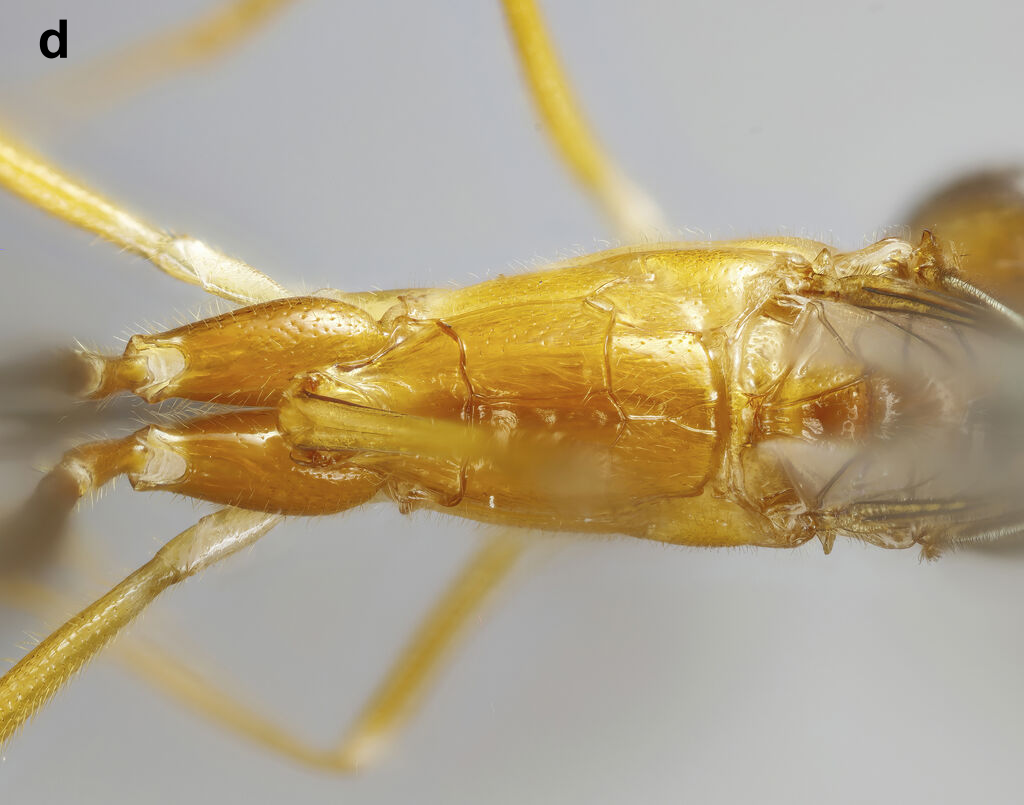
Propodeum (dorsal)

**Figure 2e.**
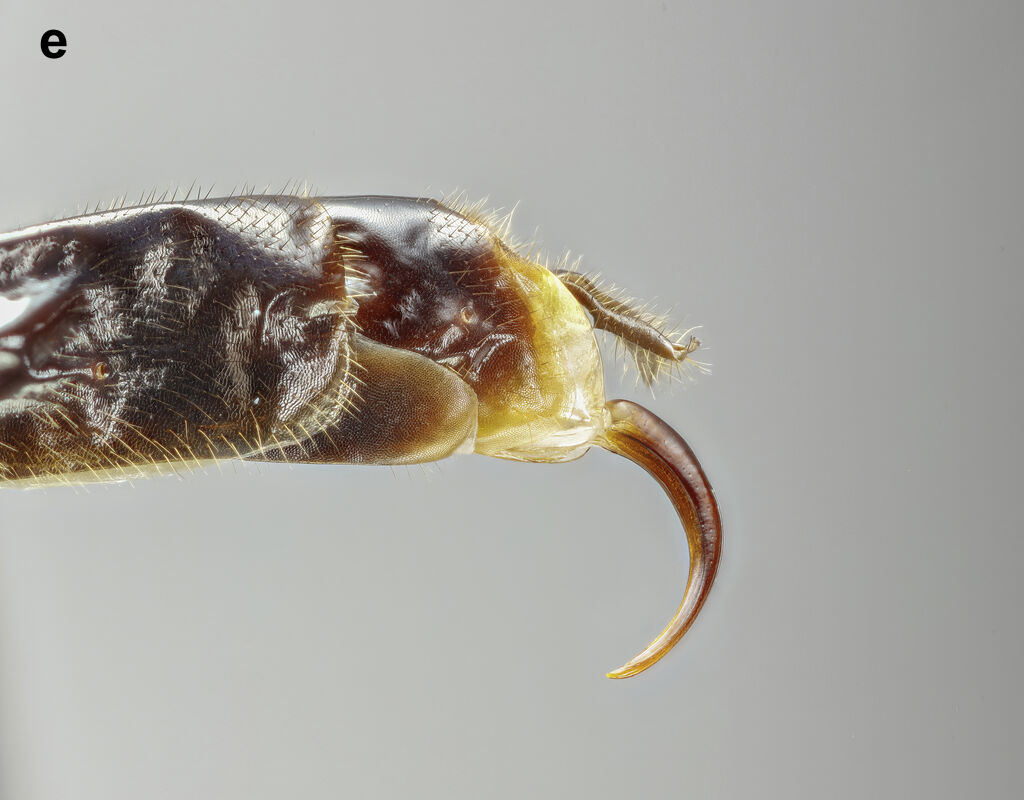
Ovipositor, lateral

**Figure 3a.**
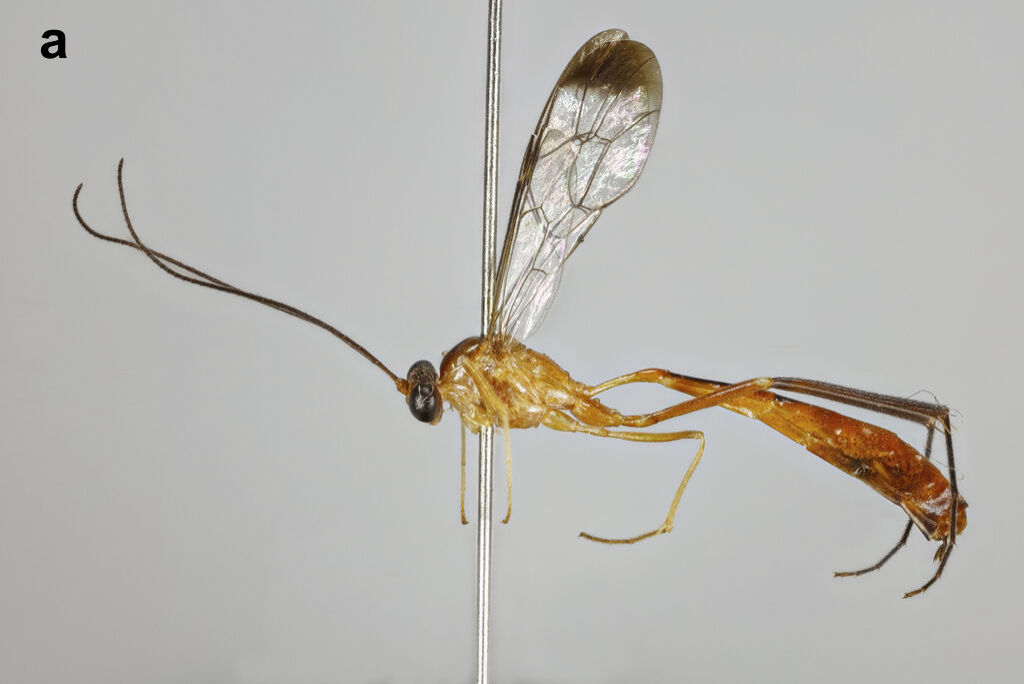
Habitus (lateral)

**Figure 3b.**
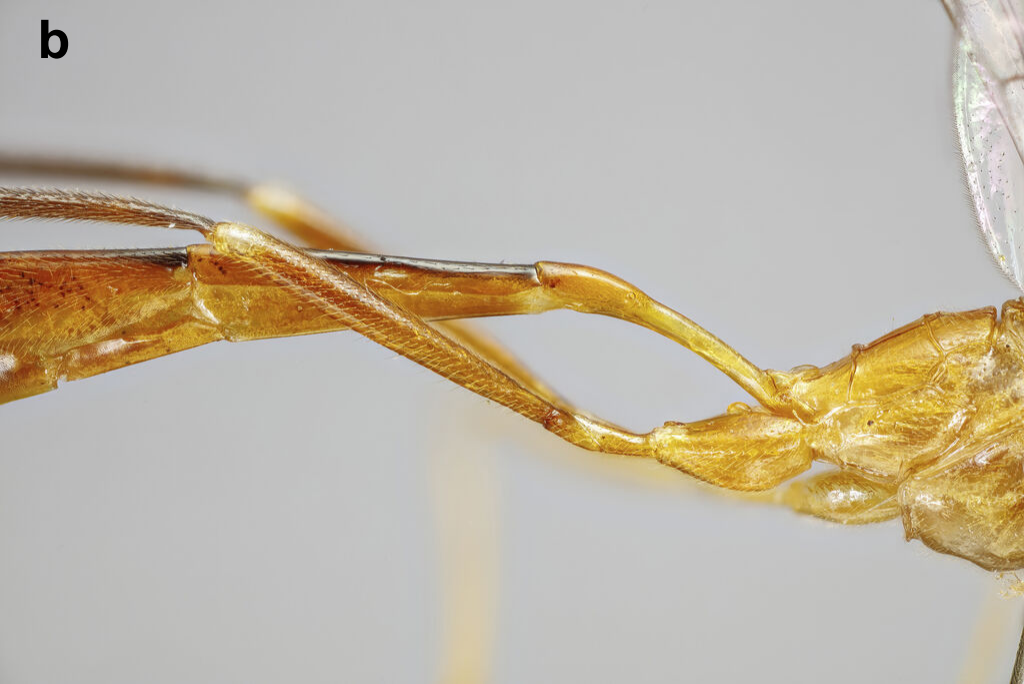
First and second tergites (lateral)

**Figure 3c.**
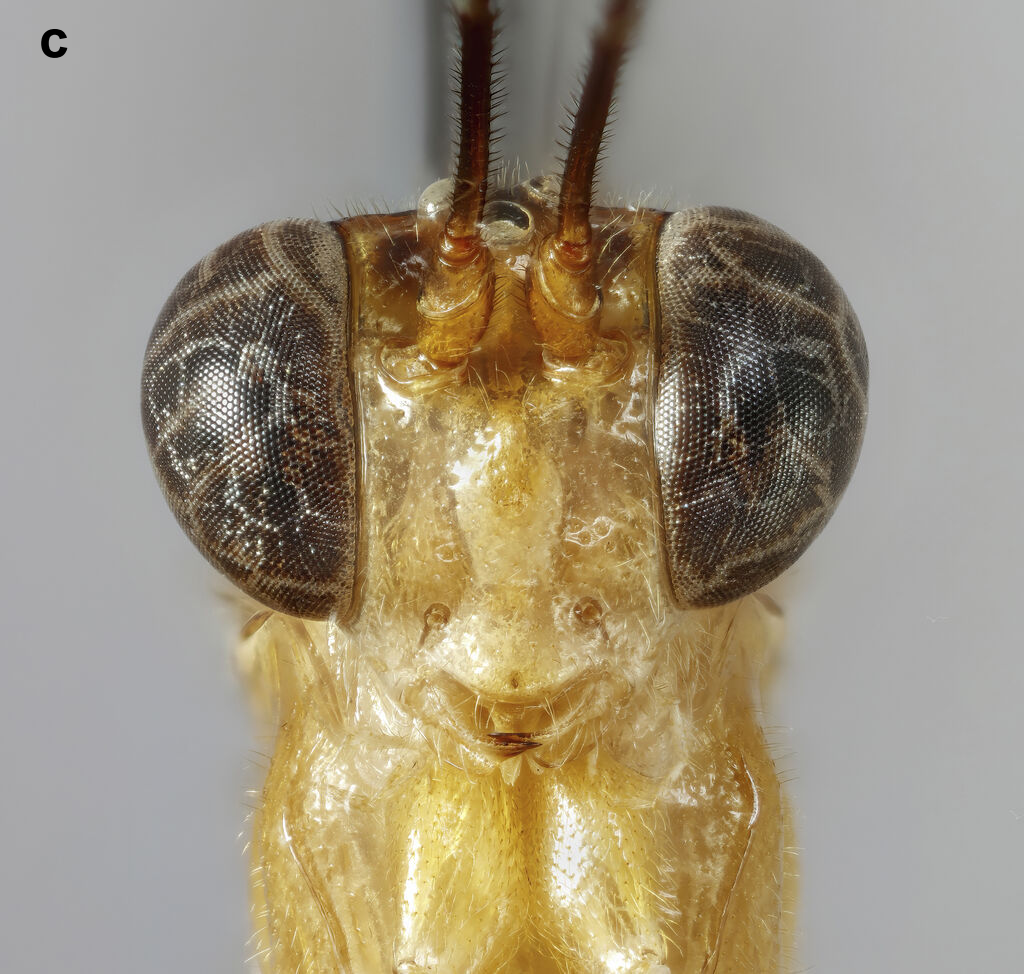
Face (anterior)

**Figure 3d.**
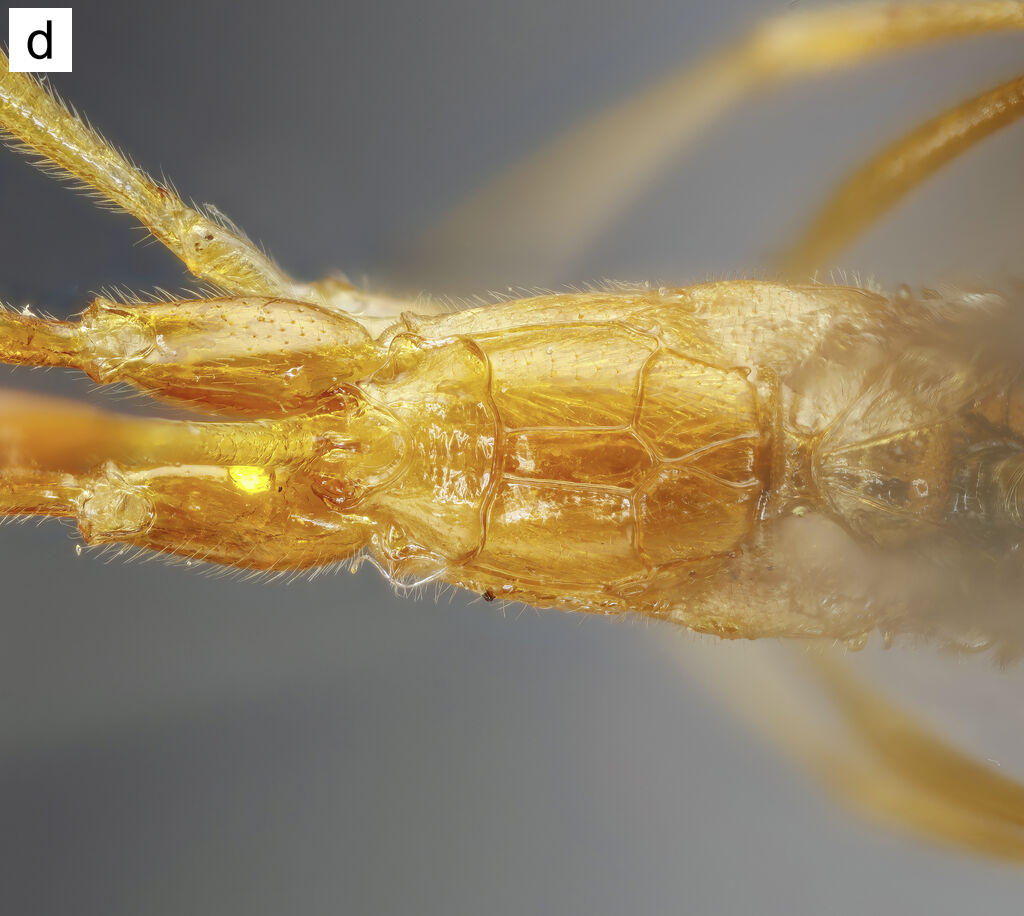
Propodeum (dorsal)
